# Levels of matrix metalloproteinase-9 and tissue inhibitor of metalloproteinase-1 are related to cardiopulmonary injury in fetal inflammatory response syndrome

**DOI:** 10.6061/clinics/2020/e2049

**Published:** 2020-11-10

**Authors:** Yiwei Yan, Lian Jiang, Mei Li, Huifen Zhang, Ying Shen, Wenhao Zhang, Wenting Zhang

**Affiliations:** Department of Pediatrics, The Fourth Hospital of Hebei Medical University, Shijiazhuang, China

**Keywords:** Preterm Infants, Fetal Inflammatory Response Syndrome, Bronchopulmonary Dysplasia, Myocardial Injury

## Abstract

**OBJECTIVES:**

To evaluate the diagnostic value of matrix metalloproteinase-9 (MMP-9), tissue inhibitor of metalloproteinase-1 (TIMP-1), and the MMP-9/TIMP-1 ratio in fetal inflammatory response syndrome (FIRS), and determine a possible association with the incidence of bronchopulmonary dysplasia (BPD) and myocardial injury.

**METHODS:**

Overall, 61 cases of preterm infants with FIRS were divided into the FIRS group 1 (≤32 weeks) and FIRS group 2 (32 to 37 weeks). Similarly, 57 cases of normal preterm infants were divided into Control group 1 and Control group 2. Levels of interleukin-6 (IL-6), MMP-9, and TIMP-1 were detected by enzyme-linked immunosorbent assay. Spearman’s linear correlation was used to analyze the relationship between dependent variables. Pathological changes were examined by hematoxylin and eosin (HE) staining and in amniotic fluid smears.

**RESULTS:**

Levels of IL-6, MMP-9, and TIMP-1, and the MMP-9/TIMP-1 ratio were significantly higher in the FIRS group than in the Control groups. IL-6 was positively correlated with MMP-9, TIMP-1, and the MMP-9/TIMP-1 ratio. Areas under the curve (AUC) of MMP-9, TIMP-1, and the MMP-9/TIMP-1 ratio were 0.92, 0.90, and 0.95, respectively. HE staining and amniotic fluid smears showed the aggregation of inflammatory cells. MMP-9, TIMP-1, and the MMP-9/TIMP-1 ratio were closely related to the incidence of BPD (≤32 weeks) and myocardial injury (<37 weeks) in preterm infants.

**CONCLUSION:**

MMP-9, TIMP-1, and the MMP-9/TIMP-1 ratio revealed a certain diagnostic value for FIRS; combined with gestational age, these parameters were effective for predicting cardiopulmonary injury.

## INTRODUCTION

Preterm birth is characterized by birth at <37 weeks of gestation and generally causes neonatal morbidity and death (1). According to the reported births in 2018, the birth rate of premature infants increased from 9.93% to 10.02% (2). Notably, intrauterine infections are the most common cause of preterm infant births, accounting for approximately 40% of the overall cases of preterm births (3). Furthermore, intrauterine infections may cause the immune system to release numerous inflammatory factors, defined as the fetal inflammatory response syndrome (FIRS).

FIRS is characterized by a plasma interleukin-6 (IL-6) level >11 pg/mL (4). Fortunately, fetal blood plasma can be obtained either by cordocentesis or from the umbilical blood plasma. Therefore, it provides an effective method for the early diagnosis of FIRS. Additional evidence has demonstrated that matrix metalloproteinases (MMPs) play important roles in pathological processes, including inflammation, cardiovascular diseases, and pulmonary diseases (5,6). However, its activity is controlled by tissue inhibitors of metalloproteinases (TIMPs). As an important member of the MMP family, MMP-9 is a major component of the basement membrane of airways (7). Therefore, the correlation between IL-6 and MMP-9, TIMP-1, and the MMP-9/TIMP-1 ratio, as well as its diagnostic value in FIRS, warrant further exploration.

FIRS corresponds to the systemic inflammatory response syndrome (SIRS) observed in adult conditions, inducing the injuries to numerous organs during the neonatal period (8). Numerous studies have increasingly focused on injuries in the fetal lung, brain, and heart (9,10) as imaging can be easily performed. As previously documented, bronchopulmonary dysplasia (BPD) and myocardial injury are common disorders in preterm infants (11,12). Previous studies have indicated that IL-6 and the MMP-9/TIMP-1 ratio are related to the pathogenesis of BPD (13,14). However, whether the MMP-9/TIMP-1 ratio is related to the pathogenesis of myocardial injury remains unclear.

The aim of this study was to evaluate the correlation between IL-6 and MMP-9, and TIMP-1, and the MMP-9/TIMP-1 ratio in the umbilical cord blood of patients with FIRS. The findings of this study may help provide a new basis for predicting health disorders in preterm newborns.

## MATERIAL AND METHODS

### Patients

In total, 118 preterm infants born at 27^+6^-36^+5^ weeks of gestation were selected from May 2014 to December 2015 at the Fourth Hospital of the Hebei Medical University. Overall, 61 infants who conformed to the diagnostic criteria of FIRS were included in the FIRS group, with the remaining infants (57 infants) included in the control groups. Among them, 33 cases in the FIRS group showed a gestational age ≤32 weeks (FIRS group 1) and 28 cases showed a gestational age between 32-37 weeks (FIRS group 2). Similarly, 25 cases in the control group showed a gestational age of ≤32 weeks (Control group 1) and 32 cases showed a gestational age between 32-37 weeks (Control group 2).

For FIRS, the main diagnostic indicators consisted of an IL-6 level>11 pg/mL in the umbilical cord blood, C-reactive protein (CRP) level>6 mg/L in the amniotic fluid, inflammatory infiltration in the placenta and umbilical cord, and accumulation of inflammatory cells in the amniotic fluid. Other indicators were used as auxiliary diagnoses, including maternal fever, heart rate>100 beats per minute, fetal heart rate>160 beats per minute, uterine tenderness, amniotic fluid odor, and a peripheral blood white blood cell count>1.5×10^10^/L. During follow-up, cases of BPD and myocardial injury were recorded separately, and the incidence of each was calculated. The protocol was approved by the Institutional Ethics Committee of the Fourth Hospital of Hebei Medical University (2020004), and informed consent was obtained from the parents of the infants.

### Sample collection

Blood samples (2-3 mL) were obtained from the umbilical vein immediately after delivery and then centrifuged at 3000 rpm for 10 min. The serum was collected and stored at -80°C. Amniotic fluid samples (2-3 mL) were obtained during delivery. After mixing, amniotic fluid samples (0.2-0.3 mL) were used to perform the smear test. The amniotic fluid samples were centrifuged at 3500 rpm for 15 min. Then, the supernatants were collected and stored at -80°C until analysis. Once the placenta was delivered, tissues such as the placenta, umbilical cord, and fetal membranes were collected under relatively sterile conditions, placed in a neutral formaldehyde solution, and stored at 4°C until analysis.

### Enzyme-linked immunosorbent assay (ELISA)

The serum concentrations of IL-6, MMP-9, and TIMP-1 were determined using double-antibody sandwich ELISA kits (Shanghai ELISA Biotech Co., Ltd., Shanghai, China), and the absorbance of the sample was measured using a microplate reader (Molecular Devices, VersaMax, California, USA). All procedures were performed according to the manufacturer’s instructions.

### Hematoxylin-eosin (HE) staining

HE staining was performed to analyze pathological changes in the placenta. Briefly, tissues were sliced into 4-μm-thick sections, deparaffinized, and stained with HE. Images were captured with a microscope (Olympus CX-21, Olympus Corp., Tokyo, Japan).

### Examination of amniotic fluid smears

In brief, amniotic fluid (0.2-0.3 mL) was placed dropwise onto a glass slide, and a uniform thin layer was formed. Then, Wright’s stain (Wenzhou Kangtai Biotechnology Company, Wenzhou, China) was added, along with distilled water, in a dropwise manner. After mixing, the smear was washed and examined microscopically.

### Follow-up

Data on hospitalization and post-discharge were collected, and preterm infants were followed up for 28 days. BPD and myocardial injury cases in the later period were recorded, and the incidence of BPD and myocardial injury was calculated.

For BPD, the diagnostic criteria were preterm infants who underwent mechanical ventilation or were treated with oxygen therapy and were still dependent on oxygen 28 days after birth. For myocardial injury, the diagnostic criteria were as follows: 1) elevated levels of myocardial enzymes; 2) elevated troponin levels; 3) abnormal electrocardiograph, including bradycardia and arrhythmia; 4) echocardiographic abnormalities, including altered heart structure, impaired systolic function, and impaired left ventricular ejection fraction.

### Statistical analysis

Statistical analyses were performed using SPSS 13.0 (IBM Corp., Chicago, USA). The quantitative data are presented as the means±standard deviations (SDs) and were analyzed by Student’s *t*-test for the comparison of data from two groups. Categorical data were analyzed using the χ^2^ test and expressed as a percentage. Spearman’s linear correlation was used to analyze the relationship between dependent variables. Differences with *p*-values<0.05 were considered statistically significant.

## RESULTS

### IL-6, MMP-9, and TIMP-1 were upregulated in the cord blood of preterm infants

The clinical characteristics of the two groups are shown in Table 1. There were no obvious differences in gestational age, sex, birth weight, and Apgar scores at 1 min, 5 min, and 10 min between the FIRS and Control groups (all *p*>0.05). Furthermore, the current study revealed that the levels of IL-6, MMP-9, and TIMP-1, and the MMP-9/TIMP-1 ratio in the FIRS group 1 with the same gestational age significantly increased compared with those in Control group 1 (all *p*<0.01). Additionally, the levels of IL-6, MMP-9, and TIMP-1, and the MMP-9/TIMP-1 ratio were higher in the FIRS group 2 than those observed in Control group 2 (all *p*<0.01) (Table 2).

Among preterm infants with FIRS, the levels of IL-6, MMP-9, and TIMP-1, and the MMP-9/TIMP-1 ratios were not statistically different between FIRS group 1 and FIRS group 2 *(p>*0.05). Additionally, the IL-6 level in Control Group 2 did not differ significantly from that in Control group 1 (*p*>0.05). However, the levels of MMP-9 and TIMP-1 in Control group 2 were significantly increased, whereas the MMP-9/TIMP-1 ratio was significantly decreased (all *p*<0.01) (Table 2).

### The correlation between IL-6 and MMP-9, TIMP-1, and the MMP-9/TIMP-1 ratio

**Figure 1:**
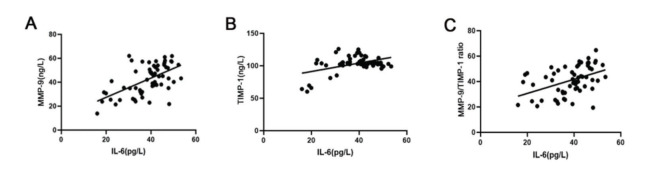
The correlation between IL-6 and MMP-9, TIMP-1, and the MMP-9/TIMP-1 ratio in preterm infants. (A) Spearman’s linear correlation results indicate that IL-6 was positively correlated with MMP-9. (B) Spearman’s linear correlation results indicate that IL-6 was positively correlated with TIMP-1; (C) Spearman’s linear correlation results indicate that IL-6 was positively correlated with the MMP-9/TIMP-1 ratio. IL-6, interleukin 6; MMP-9, matrix metallopeptidase 9; TIMP-1, tissue inhibitor matrix metalloproteinase 1.

Based on Spearman’s linear correlation analysis, IL-6 was positively correlated with the levels of MMP-9 and TIMP-1 and the MMP-9/TIMP-1 ratio (p_MMP-9_<0.01, r_MMP-9_=0.58; p_TIMP-1_<0.01, r_TIMP-1_=0.45; p_MMP-9/TIMP-1 ratio_<0.01, r_MMP-9/TIMP-1 ratio_=0.44) (Figure 1A-C).

### The diagnostic value of cytokines in predicting FIRS

The critical values of MMP-9 levels, TIMP-1 levels, and the MMP-9/TIMP-1 ratio for predicting FIRS are shown in Table 3. The area under the curve (AUC) of MMP-9, TIMP-1, and the MMP-9/TIMP-1 ratio were 0.92, 0.90, and 0.95, respectively. The sensitivity and specificity of these indicators were above 90%.

### Histological examination of placental tissues in preterm infants

**Figure 2:**
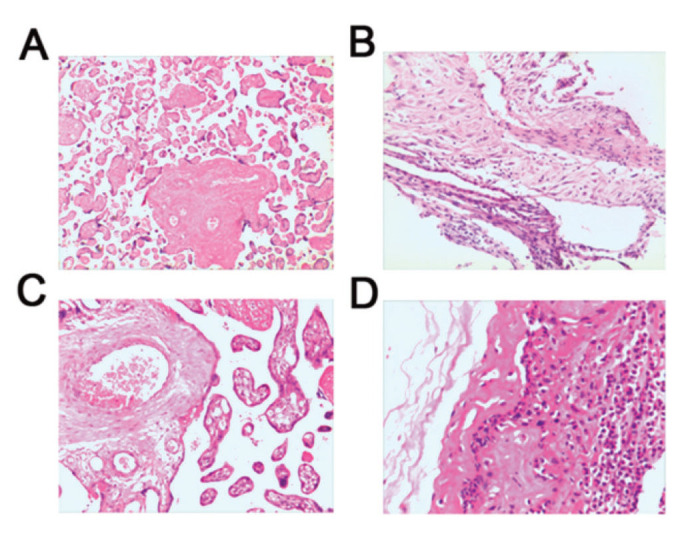
Histological changes in placental tissues obtained from preterm infants were examined by HE staining. (A) Normal placental tissue from preterm infants demonstrates no obvious pathological change (×200). (B) Chorionic membrane tissue demonstrates mild inflammatory cell infiltration (×200). (C) Acute inflammatory placental tissues show congestion and edema, with aggregated neutrophils (×200). (D) Chronic inflammatory placental tissues show necrosis, proliferation, neutrophils, lymphocytes, and monocytes (×200). HE, hematoxylin and eosin.

No pathological changes were observed in the placenta of subjects from the Control groups (Figure 2A). Chorioamnionitis was the most prevalent lesion observed in the chorion, and acute and chronic inflammatory cell infiltration was documented (Figure 2B). However, pathological changes in the placenta of subjects from the FIRS group showed inflammatory cell accumulation, bleeding, and necrosis. A large number of neutrophils were aggregated in the tissues showing acute inflammation, along with congestion and edema. Inflammatory cells such as neutrophils, lymphocytes, and monocytes were observed in the tissues showing chronic inflammation, with necrosis and proliferation (Figure 2C, 2D).

### Examination of amniotic fluid smears in preterm infants

**Figure 3:**
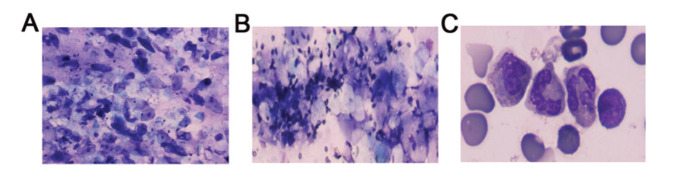
Examination of amniotic fluid smears of preterm infants. (A) The amniotic fluid smear for normal preterm infants mainly shows exfoliated epithelial cells and a small number of impurities (×400). (B) The amniotic fluid smear for preterm infants with FIRS shows inflammatory cells, mostly including neutrophils and a few aggregated lymphocytes (×400). (C) Images of the amniotic fluid smear for preterm infants with FIRS were captured using oil immersion microscopy. FIRS, fetal inflammatory response syndrome.

As shown in Figure 3B, 3C, the amniotic fluid smears revealed inflammatory cells, including a large number of neutrophils and few lymphocytes, aggregated in the samples from subjects in the FIRS group. However, the smear samples of subjects from the Control groups mainly showed exfoliated epithelial cells and minimal impurities (Figure 3A).

### Follow-up

During follow-up, the incidence of BPD and myocardial injury was higher in FIRS group 1 than in Control group 1 (*p*<0.05). Additionally, the incidence of myocardial injury was higher in FIRS group 2 than in Control group 2 (*p*<0.05), whereas no significant difference was observed in the incidence of BPD between the two groups (*p*>0.05) (Tables 4 and 5).

Among preterm infants with FIRS, the incidence of BPD was higher in FIRS group 1 than in FIRS group 2 (*p*<0.05), with no significant difference in the incidence of myocardial injury being observed between the two groups (*p*>0.05). The incidence of BPD and myocardial injury did not significantly differ between Control group 1 and Control group 2 (*p*>0.05) (Tables 4 and 5).

## DISCUSSION

Preterm birth is classified as extremely preterm (<28 weeks), very preterm (28 to 32 weeks), and moderate to late preterm (32 to 37 weeks) based on the gestational age (15). It has been reported that preterm birth is closely related to FIRS (16). In the current study, the levels of IL-6, MMP-9, and TIMP-1, and the MMP-9/TIMP-1 ratio in the umbilical cord blood of infants presenting FIRS (gestational age of ≤32 weeks and 32 to 37 weeks) were upregulated, and IL-6 levels were positively correlated with the MMP-9 and TIMP-1 levels and the MMP-9/TIMP-1 ratio. Additionally, these inflammatory indicators presented a certain diagnostic value for FIRS, which combined with the gestational age, were effective indicators for predicting the incidence of BPD and myocardial injury.

A previous review has summarized the association between the inflammatory response and premature birth, which indicates that IL-6 (>11 pg/mL) is considered an independent risk factor for the subsequent development of major neonatal morbidity (17). Furthermore, investigations regarding cytokines in the umbilical cord blood aid in the diagnosis of FIRS. For example, Rangel-Frausto et al. (18) have demonstrated that elevated IL-6 levels precede the onset of preterm birth. A similar study has revealed that the inflammatory marker IL-6 is closely associated with tumor necrosis factor (TNF)-α in the umbilical cord blood at 22-34 weeks of gestation, and IL-6 and TNF-α are of notable value for the diagnosis of FIRS (19). In our study, the levels of MMP-9 and TIMP-1 and the MMP-9/TIMP-1 ratio were higher in the FIRS groups (FIRS group 1 and FIRS group 2) than in the Control groups (Control group 1 and Control group 2), consistent with the reported IL-6 levels. Additionally, the levels of MMP-9 and TIMP-1 and the MMP-9/TIMP-1 ratio revealed a good diagnostic value in predicting the incidence of FIRS. These results indicated that MMP-9, TIMP-1, and the MMP-9/TIMP-1 ratio may be predictive indicators for FIRS.

It is well known that inflammatory responses mediated via IL-6, IL-1β, and TNF-α stimulate the secretion of MMPs (20,21). MMPs include collagenases (MMP-1, MMP-8, and MMP-13), stromelysins (MMP-3, MMP-7, and MMP-10), and gelatinases (MMP-2 and MMP-9). MMP-9 is known as gelatinase-B, which is a type 4 collagenase secreted by leukocytes and is related to fetal membrane damage (22). A previous study has reported that MMP-9 levels appear to be dependent on gestational age, with the highest levels observed at 33-36 weeks of gestation in premature infants with no BPD, and the TIMP-1 level was highest in full-term neonates (23). Likewise, Bednarek et al. (24) have reported that the MMP-9 level is dependent on gestational age in premature infants in the control group. Surprisingly, our study found that the levels of MMP-9 and TIMP-1 in Control group 2 were higher than those in Control group 1; however, the MMP-9/TIMP-1 ratio in Control group 2 was lower than that in Control group 1. This was attributed to the increased MMP-9 level, which was significantly lower than the TIMP-1 level in Control group 2. Similarly, the MMP-9/TIMP-1 ratio was slightly lower in FIRS group 2 than in FIRS group 1. A previous study has demonstrated that the MMP-9/TIMP-1 ratio is negatively correlated with gestational age, which is consistent with the findings of our study, indicating that lung development gradually matures with increasing gestational age (14,25). Additionally, a previous study has indicated that the MMP-9/TIMP-1 ratio is closely correlated with the duration of oxygen supplementation, suggesting the extent of cardiopulmonary injury (14). In the current study, the incidence of BPD and myocardial injury was slightly lower in Control group 2 than in Control group 1; however, no significant differences were observed, which is consistent with the trend of the MMP-9/TIMP-1 ratio. Furthermore, a high MMP-9/TIMP-1 ratio is considered a risk factor for lung damage (26). Therefore, the abovementioned reports suggest that the levels of MMP-9 and TIMP-1 and the MMP-9/TIMP-1 ratio appeared to be dependent on gestational age in premature infants in Control groups.

In addition, the levels of MMP-9 and TIMP-1 are upregulated in premature infants with BPD and encephalopathy, which has been previously confirmed (23,24). In the current study, the incidence of both BPD and myocardial injury were higher in FIRS group 1 than in Control group 1. However, the incidence of BPD did not significantly differ between FIRS group 2 and Control group 2. These results suggest that BPD may be closely correlated with gestational age. Our results were consistent with those reported by Mittendorf et al. (13), indicating that gestational age and low birth weight can act as major risks for BPD. Additionally, the current study revealed that the incidence of BPD in FIRS group 1 was higher than that in FIRS group 2. All obtained data suggested that the levels of MMP-9 and TIMP-1, combined with gestational age, may be novel indicators for predicting the incidence of BPD and myocardial injury.

Several reports have demonstrated that the MMP-9/TIMP-1 ratio is associated with the development of some diseases in newborn infants, including bronchiectasis and lung infection (27,28). In terms of BPD, Fukunaga et al. (14) have reported that in infants at a gestational age <30 weeks, the MMP-9/TIMP-1 ratio in the cord blood is related to the development of BPD. A subsequent study has reported that the MMP-9/TIMP-1 ratio plays an important role in the progression of BPD, and it was observed that Mn (III) tetrakis (4-benzoic acid) porphyrin chloride can effectively decrease the MMP-9/TIMP-1 ratio in a rat model (29). Therefore, the MMP-9/TIMP-1 ratio, combined with the gestational age, can be used to predict the possibility of BPD in premature infants with FIRS. Furthermore, we observed that the incidence of myocardial injury in the FIRS groups was higher than that in the Control groups. In the present study, the relationship between the MMP-9/TIMP-1 ratio and myocardial injury was in accordance with the findings of previous investigations reporting that the MMP-9/TIMP-1 ratio is related to brain injury (24). These results suggested that the MMP-9/TIMP-1 ratio was closely associated with the incidence of BPD (gestational age≤32 weeks) and myocardial injury (gestational age<37 weeks) in preterm infants.

## CONCLUSIONS

In FIRS, the levels of IL-6, MMP-9, and TIMP-1, and the MMP-9/TIMP-1 ratio in the umbilical cord blood were upregulated, and IL-6 levels were positively correlated with the levels of MMP-9 and TIMP-1 and the MMP-9/TIMP-1 ratio. Furthermore, these inflammatory indicators presented a certain diagnostic value in FIRS; combined with gestational age, these markers were effective in predicting the incidence of BPD and myocardial injury.

## AUTHOR CONTRIBUTIONS

Jiang L conceived and designed this study; Zhang WH and Zhang HF provided materials and samples; Yan YW, Zhang WT, and Li M participated in data collection, analysis, and interpretation of the results. Yan YW wrote the manuscript. Shen Y, and Jiang L provided administrative support.

## Figures and Tables

**Table 1:** General characteristics of the two groups.

	FIRS group (n=61)	Control group (n=57)	t/F value	*p*-value
Male/Female (n)	32/29	30/27	0.00	0.99
Gestational age (weeks)	29.35±1.44	29.29±1.51	0.22	0.83
Birth weight (g)	1489±178	1482±169	0.22	0.83
Apgar score at 1 min	7.28±1.42	7.32±1.34	0.16	0.88
Apgar score at 5 min	8.39±1.52	8.43±1.45	0.15	0.88
Apgar score at 10 min	9.01±1.26	9.12±1.32	0.46	0.64

FIRS, Fetal inflammatory response syndrome.

**Table 2:** Levels of IL-6, MMP-9, and TIMP-1, and the MMP-9/TIMP-1 ratio in the different groups.

	IL-6 (pg/L)	MMP-9 (ng/L)	TIMP-1 (ng/L)	MMP-9 / TIMP-1 ratio (%)
Control group 1	4.39±0.99	1.68±0.46	12.57±2.00	13.77±4.37
FIRS group 1	42.68±6.98**	48.48±10.2**	106.07±6.61**	45.95±10.96**
Control group 2	4.51±1.03	4.80±1.51**	60.93±7.37**	7.92±2.45**
FIRS group 2	33.63±8.77##	34.94±11.08##	99.97±17.38##	35.04±9.00##

FIRS, Fetal inflammatory response syndrome; IL-6, interleukin 6; MMP-9, matrix metallopeptidase 9; TIMP-1, tissue inhibitor matrix metalloproteinase 1;

** indicates *p*<0.01 compared with Control group 1;

## indicates *p*<0.01 compared with Control group 2.

**Table 3:** The diagnostic value of cytokines for predicting FIRS.

	Cutoff value	Sensitivity (%)	Specificity (%)	95% CI	AUC
MMP-9	21.61	0.98	0.91	0.86-0.98	0.92
TIMP-1	78.76	0.94	0.91	0.83-0.97	0.90
MMP-9 / TIMP-1 ratio	22.50	0.96	0.90	0.90-0.99	0.95

FIRS, Fetal inflammatory response syndrome; CI, Confidence interval; AUC, Area under the curve; IL-6, interleukin 6; MMP-9, matrix metallopeptidase 9; TIMP-1, tissue inhibitor matrix metalloproteinase 1.

**Table 4:** The incidence of BPD in the different groups.

	Occurrence (n)	Nonoccurrence (n)	Total (n)	Rate (%)
Control group 1	2	23	25	8.00
FIRS group 1	10	23	33	30.30*
Control group 2	0	32	32	0
FIRS group 2	1	27	28	3.57& &

BPD, Bronchopulmonary dysplasia; FIRS, Fetal inflammatory response syndrome;

* indicates *p*<0.05 compared with Control group 1;

&& indicates *p*<0.01 compared with FIRS group 1.

**Table 5:** The incidence of myocardial damage in the different groups.

	Occurrence (n)	Nonoccurrence (n)	Total (n)	Rate (%)
Control group 1	2	23	25	8.00
FIRS group 1	11	22	33	33.33*
Control group 2	1	31	32	3.13
FIRS group 2	6	22	28	21.43#

FIRS, Fetal inflammatory response syndrome;

* indicates *p*<0.05 compared with Control group 1;

# indicates *p*<0.05 compared with Control group 2.
